# Thermography methods to assess stomatal behaviour in a dynamic environment

**DOI:** 10.1093/jxb/erz573

**Published:** 2020-01-08

**Authors:** Silvere Vialet-Chabrand, Tracy Lawson

**Affiliations:** 1 School of Life Sciences, University of Essex, Colchester, UK; 2 Research School of Biology, Australian National University, Australia

**Keywords:** Energy balance, patchy response, stomatal conductance, stress indices, thermal imaging, transpiration

## Abstract

Although thermography allows rapid, non-invasive measurements of large numbers of plants, it has not been used extensively due to the difficulty in deriving biologically relevant information such as leaf transpiration (*E*) and stomatal conductance (*g*_sw_) from thermograms. Methods normalizing leaf temperature using temperatures from reference materials (e.g. with and without evaporative flux) to generate stress indices are generally preferred due to their ease of use to assess plant water status. Here, a simplified method to solve dynamic energy balance equations is presented, which enables the calculation of ‘wet’ and ‘dry’ leaf temperatures in order to derive stress indices, whilst providing accurate estimates of *E* and *g*_sw_. Comparing stress indices and gas exchange parameters highlights the limitation of stress indices in a dynamic environment and how this problem can be overcome using artificial leaf references with known conductance. Additionally, applying the equations for each pixel of a thermogram to derive the rapidity of stomatal response over the leaf lamina in wheat revealed the spatial heterogeneity of stomatal behaviour. Rapidity of stomatal movements is an important determinant of water use efficiency, and our results showed ‘patchy’ responses that were linked to both the spatial and temporal response of *g*_sw_.

## Teaser

New methodology enables transpiration and stomatal conductance to be derived from thermograms which were compared against thermal indices, revealing spatial patterns in rapid stomatal responses that limit plant performance.

## Introduction

Plant phenotyping, the quantitative description of anatomical, ontogenetical, physiological, and biochemical properties of plants (Walter *et al.*, 2015), is key for assessing genetic diversity and identifying targets for genetic manipulation to improve plant performance and yield (Fahlgren *et al.*, 2015; Prado *et al.*, 2018). Leaf stomatal conductance to water vapour (*g*_sw_) is an important physiological trait for phenotyping, as stomatal behaviour impacts photosynthetic CO_2_ uptake, transpiration, and temperature from the leaf to the canopy, all of which influence plant yield and water status (Fischer and Rebetzke, 2018). Although very precise, the scalability of leaf gas exchange methods to estimate *g*_sw_ is limited and is becoming a major technical bottleneck for large-scale studies (Araus and Cairns, 2014; Rischbeck *et al.*, 2017; Prado *et al.*, 2018). In contrast, thermography allows rapid, non-invasive measurements of large numbers of plants; however, its biological interpretation is more challenging (Jones *et al.*, 2018).

In recent years, the majority of studies assessing plant water status have focused on methods to normalize leaf temperature using temperatures from reference materials due to their ease of use (Grant *et al.*, 2016; Maes *et al.*, 2016; Yu *et al.*, 2017; Bian *et al.*, 2019). The values obtained through use of reference material temperatures with and without evaporative flux to determine the level of evaporative cooling are generally referred to as ‘stress indices’ (Jones, 2013, 2018; Prashar and Jones, 2016). One of the first reported was the crop water stress index (CWSI; Jackson *et al.*, 1981; Idso, 1982) that normalizes leaf temperature by comparing measurements from plants under well-watered and water-stressed conditions in a similar environment. Such an approach is difficult to apply in the field; therefore Jones (1999) proposed using a ‘wet’ (transpiring with infinite surface conductance) and a ‘dry’ (non-transpiring) reference material (e.g. green felt) as a replacement for reference plants. This stress index has since been used to evaluate levels of plant water stress in the field (Maes *et al.*, 2016) and has also been included in algorithms for automatic plant watering systems to reduce production costs and resource use, whilst maintaining yield (Osroosh *et al*., 2015, 2016).

In theory, diurnal estimation of the transpiration rate (*E*) and *g*_sw_ can more accurately identify phenotypic differences in plant water use and regulation than stress indices. Therefore, there is a need to develop easy to use methods to assess *E* and *g*_sw_ in the field and compare performance against stress indices. To derive *E* and *g*_sw_ from thermograms, leaf energy balance equations can be used to describe the energy exchanged between the leaf and the environment. These equations provide a mechanistic background that simplifies and improves the biological interpretation, but increases the level of complexity of data processing. Therefore, despite all efforts to simplify their application, energy balance equations are still not widely applied or standardized (Leinonen *et al.*, 2006; Guilioni *et al.*, 2008; Maes *et al.*, 2016; Jones *et al.*, 2018). Recently, a new method that enables the prediction of the thermal kinetics of any object using a unique reference material has been published and has opened up new opportunities to analyse thermograms (Vialet-Chabrand and Lawson, 2019). In general, reference materials used to determine thermal indices do not fully mimic leaf (thermal and optical) properties, leading to potential errors in calculated values. An easier approach is to predict the ‘dry’ and ‘wet’ reference temperatures using energy balance, as suggested by Grant *et al.* (2016). The model developed by Vialet-Chabrand and Lawson (2019) suggested that any reference material could be used to predict the ‘dry’ and ‘wet’ reference temperatures. This approach could enable the calculation of temperature stress indices whilst estimating *E* and *g*_sw_ using the same set-up. A limitation in the original approach by Vialet-Chabrand and Lawson (2019) was that *g*_sw_ was inferred using a sigmoidal model and was limited to monitoring responses to step changes in light intensity. In this study, a simplified method to calculate *E* and *g*_sw_ is presented and the results compared with those from stress indices.

Leaves frequently exhibit spatial heterogeneity of *g*_sw_, where areas or ‘patches’ of stomata have different apertures from those of adjacent regions (Lawson *et al.*, 1998; Lawson and Weyers, 1999; Mott *et al.*, 1999; Prytz *et al.*, 2003; West *et al.*, 2005; McAusland *et al.*, 2013), resulting in spatial patterns of *g*_sw_ and net CO_2_ assimilation (*A*) that are not always coordinated (Lawson and Weyers, 1999; Hanson *et al.*, 2013; McAusland *et al.*, 2013; Matthews *et al.*, 2017), impacting overall leaf *A* (Cheeseman, 1991; Weyers *et al.*, 1997; Weyers and Lawson, 1997). Spatial patterns of stomatal density may explain some of the variation in *g*_sw_ in different species (Smith *et al.*, 1989; Poole *et al.*, 1996; Weyers *et al.*, 1997; Lawson and Weyers, 1999; Lawson *et al.*, 2002). Spatial organization of veins and stomata has also been suggested as an important factor contributing to patchy stomatal behaviour by impacting the water supplied to the stomata during responses to environmental cues (Lawson *et al.*, 1998; Lawson and Weyers, 1999; Fiorin *et al.*, 2016). Stomatal density, size, and vein density all influence temporal responses of *g*_sw_ to step changes in light intensity (Drake *et al.*, 2013; Lawson and Blatt, 2014; Raven, 2014) with large variations between species (McAusland *et al.*, 2016). Despite the importance of temporal response of *g*_sw_ in the determination of water use (McAusland *et al.*, 2016; Taylor and Long, 2017) and yield (Qu *et al.*, 2016; Adachi *et al.*, 2019), little is known on how stomatal responses are coordinated over the leaf lamina and how the heterogeneous distribution of stomata impacts temporal responses. Here, we demonstrate the use of a simplified method for deriving *g*_sw_ using dynamic energy balance and its application to map the rapidity of stomatal responses over the leaf lamina to visualize the spatial and temporal patterns of *g*_sw_.

### Theory of leaf temperature interpretation

#### Temperature stress indices

Indices used in this study were defined following Jones, (1999) using a ‘wet’ and ‘dry’ leaf temperature to normalize temperature readings. The CWSI was calculated as follows:

$$\begin{array}{l} \begin{array}{l} CWSI=\frac{T_{leaf}-T_{dry}}{T_{wet}-T_{dry}} \end{array} \end{array}$$ (1)

and the conductance index (*I*_g_) is defined as:

$$\begin{array}{l} \begin{array}{l} I_{g}=\frac{T_{dry}-T_{leaf}}{T_{leaf}-T_{wet}} \end{array} \end{array}$$ (2)

Previous studies have used a leaf covered in petroleum jelly as a ‘dry’ reference, although caution should be applied as there is no guarantee that the thermal and optical properties of the leaf are not altered. In these studies, the ‘wet’ temperature reference has been estimated using a leaf spray with water for short-term estimations or transpiring reference material (e.g. felt or filter paper) for longer term measurements. An alternative approach is to predict leaf ‘wet’ and ‘dry’ temperature using leaf energy balance equations (Grant *et al.*, 2016).

#### Dynamic leaf energy balance

Using the temperature of a non-transpiring reference material (*T*_2_, *K*) with known optical and thermal properties, it is possible to predict the temperature kinetic (*T*_1_, *K*) of any transpiring material using the equation proposed by Vialet-Chabrand and Lawson (2019):

$$\begin{array}{l} \begin{array}{l} \frac{dT_{1}}{dt}=\frac{\begin{array}{c} k_{2}\frac{dT_{2}}{dt}-I_{s}\left(\alpha_{2}-\alpha_{1}\right)-\left(\varepsilon_{2}-\varepsilon_{1}\right)L_{d}\\ +2\sigma \left(\varepsilon_{2}{T_{2}}^{4}-\varepsilon_{1}{T_{1}}^{4}\right)\\ +2\rho C_{s}\left[g_{bh2}\left(T_{2}-T_{air}\right)-g_{bh1}\left(T_{1}-T_{air}\right)\right]-\lambda E_{1} \end{array}}{k_{1}} \end{array} \end{array}$$ (3)

with *k*_1_ and *k*_2_ the amount of energy per unit area required to change the temperature of the material by 1K (J m^−2^ K^−1^), α_1_ and α_2_ the absorbance to short-wave radiations (incident and diffuse; *I*_s_, W m^−2^), σ the Stefan–Boltzmann constant (W m^−2^ K^−4^), ε _1_ and ε _2_ the emissivity, *L*_d_ the long-wave radiation (W m^–2^) received from the surrounding environment (e.g. soil, wall), g_bh1_ and *g*_bh2_ the one-sided boundary layer conductance to heat transfer (m s^−1^), ρ the air density (kg m^−3^), *C*_s_ the specific heat capacity of humid air (J kg^−1^ K^−1^), *T*_air_ the air temperature (K), λ the latent heat of evaporation of water (J kg^−1^), and *E*_1_ the evaporative flux (kg m^−2^ s^−1^). *L*_d_ can be approximated by measuring the temperature of crumpled aluminium foil (which reflects infrared from the surrounding) using a thermal camera with an emissivity set to 1 to calculate the energy received (σ*T*^4^). In this study, the reference and leaf materials had similar emissivity, and *L*_d_ was not included in the equation. Equation 1 was solved using an ordinary differential equation (ODE) solver (in R using the package deSolve) as described in Vialet-Chabrand and Lawson (2019) and was used to predict the temperature kinetics of a non-transpiring leaf (*T*_dry_) by removing the evaporative cooling, *E*_1_ from the energy budget. Terms $\frac{dT_{1}}{dt}$ and $\frac{dT_{2}}{dt}$ represent the first derivative of the temperature kinetics and can be estimated using a smoothed spline fitted on the observed data (see Vialet-Chabrand and Lawson, 2019). This approach can produce unstable results depending on data noise and environmental fluctuations; however, $k_{1}\frac{dT_{1}}{dt}$ and $k_{2}\frac{dT_{2}}{dt}$ have a relatively low impact on thermal kinetic if the values of *k*_1_ and *k*_2_ remain close to each other and low, ensuring rapid response of the material to environmental variations.

Reorganizing the equation allows the evaporative flux to be estimated:

$$\begin{array}{l} \begin{array}{l} E_{1}=\frac{\begin{array}{c} k_{2}\frac{dT_{2}}{dt}-k_{1}\frac{dT_{1}}{dt}-I_{s}\left(\alpha_{2}-\alpha_{1}\right)\\ +2\sigma \left(\varepsilon_{2}{T_{2}}^{4}-\varepsilon_{1}{T_{1}}^{4}\right)\\ +2\rho C_{s}\left[g_{bh2}\left(T_{2}-T_{air}\right)-g_{bh1}\left(T_{1}-T_{air}\right)\right] \end{array}}{\lambda }\; \end{array} \end{array}$$ (4)

For a leaf, *E*_1_ in Equation 3 can be replaced by (Gates, 1966):

$$\begin{array}{l} \begin{array}{l} E_{1}=0.018\frac{1}{\frac{1}{g_{bw1}}+\frac{1}{g_{sw1}}}\left(\frac{e_{s}-e_{a}}{P_{atm}}\right) \end{array} \end{array}$$ (5)

with 0.018 the molecular weight of water (kg mol^−1^), *P*_atm_ the atmospheric pressure (Pa), *R* the gas constant (8.3145 J mol^−1^ K^−1^), *T*_leaf_ the leaf temperature (K), *e*_s_ the leaf internal vapour pressure, and *e*_a_ the air vapour pressure (Pa) (with VPD=*e*_s_–*e*_a_, with the assumption that *e*_s_ is at saturation), *g*_bw1_ the boundary layer conductance to water vapour (mol m^−2^ s^−1^, with $g_{bw1}=\frac{2\;P_{atm}}{0.92RT_{leaf}}g_{bh1}$), and *g*_sw1_ stomatal conductance to water vapour (mol m^−2^ s^−1^). Combining Equations 3 and 5, it is possible to calculate the temperature kinetics for any combination of *g*_sw1_ and *g*_bw1_. If *g*_sw1_ is assumed to be infinite and only *g*_bw1_ is kept in Equation 3, it is possible to calculate the temperature kinetics of a ‘wet’ leaf (*T*_wet_), similarly to wet reference material (e.g. felt) generally used to calculate indices. *T*_dry_ and *T*_wet_ provide the minimum and maximum boundary temperature required to calculate CWSI (Equation1) and *I*_g_ (Equation 2) whilst taking into account the thermal and optical properties of the leaf.

Reorganizing Equation 3 and replacing *E*_1_ by Equation 5 enables calculation of *g*_sw1_ (mol m^−2^ s^−1^) in two steps. First, the total conductance to water vapour (*g*_tw1_, mol m^−2^ s^−1^) is calculated:

$$\begin{array}{l} \begin{array}{l} g_{tw1}=\frac{\begin{array}{c} k_{2}\frac{dT_{2}}{dt}-k_{1}\frac{dT_{1}}{dt}-I_{s}\left(\alpha_{2}-\alpha_{1}\right)\\ +2\sigma \left(\varepsilon_{2}{T_{2}}^{4}-{\;\varepsilon }_{1}{T_{1}}^{4}\right)\\ +2\rho C_{s}\left[g_{bh2}\left(T_{2}-T_{air}\right)-g_{bh1}\left(T_{1}-T_{air}\right)\right] \end{array}}{\lambda 0.018\left(\frac{e_{s}-e_{a}}{P_{atm}}\right)}\; \end{array} \end{array}$$ (6)

Then *g*_sw1_ is derived by retrieving *g*_bw1_ from *g*_tw1_:

$$\begin{array}{l} \begin{array}{l} g_{sw1}=\frac{1}{\frac{1}{g_{tw1}}-\frac{1}{g_{bw1}}} \end{array} \end{array}$$ (7)

In contrast to Equation 3 that was solved using an ODE solver, Equations 4, 5, and 6 were solved analytically using smoothed spline functions (‘smooth.spline’ function from R) to estimate the derivatives d*T*_1_/d*t* and d*T*_2_/d*t* at the required time points. This is a simplification that allows *g*_sw_ to be determined under any conditions, compared with the original approach by Vialet-Chabrand and Lawson (2019) that was limited to sigmoidal changes in *g*_sw_ only.

#### Boundary layer conductance

**Fig. 1.**
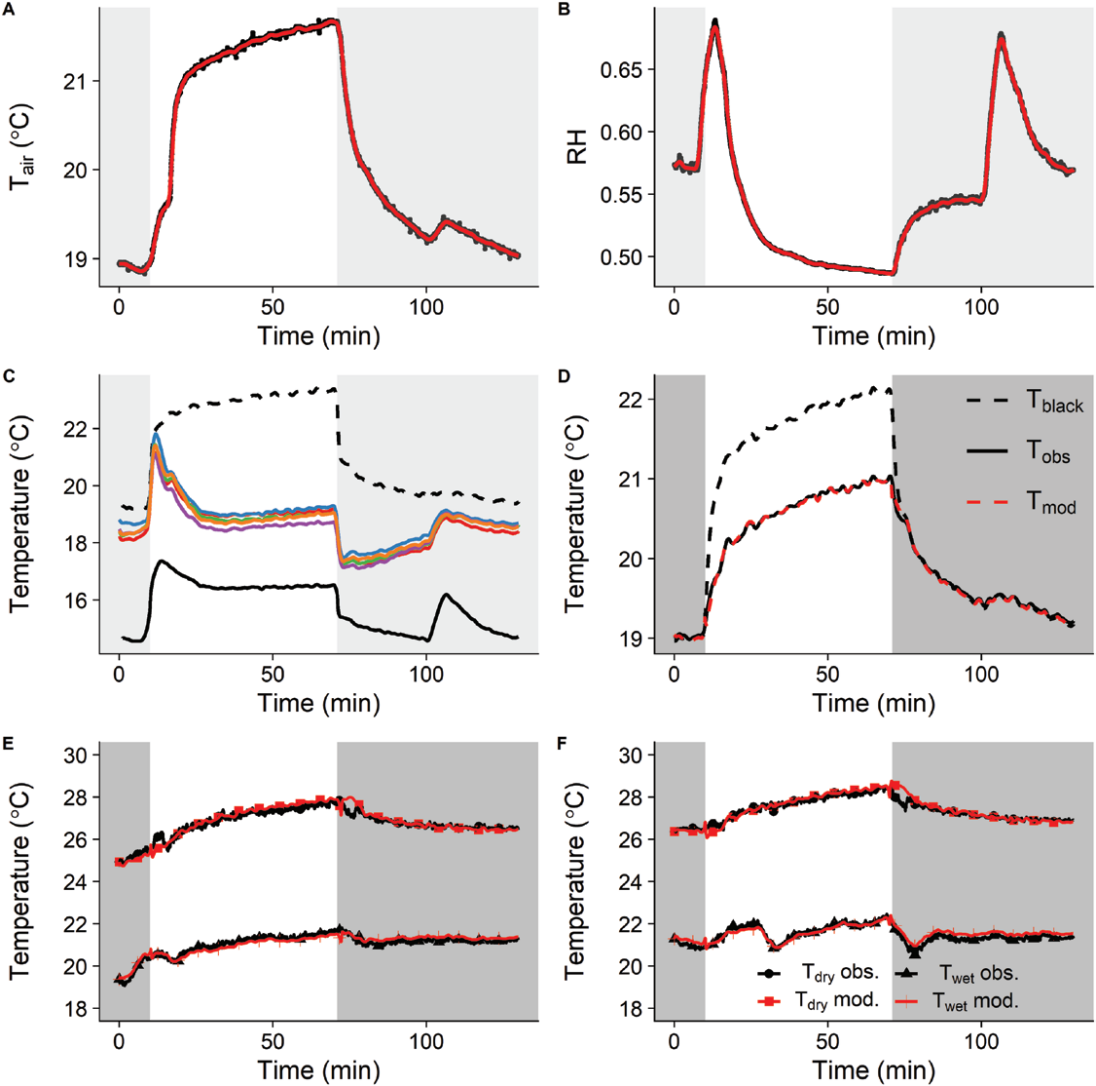
Environmental conditions and leaf temperature kinetics observed in wheat (Paragon) after a step increase in light intensity from dark to light (0 to 440 µmol m^−2^ s^−1^, 1 h) and a step decrease from light to dark (1 h). (A) Relative humidity (RH). (B) Air temperature (*T*_air_). (C) Temporal kinetic of leaf temperature (leaves in different colours) and corresponding predictions for a non-transpiring (black dashed line) and an unlimited conductance leaf (black continuous line). (D) Modelled (*T*_mod_) and observed (*T*_obs_) temperature of white reference material using black reference material temperature (*T*_black_) used to validate boundary layer estimates. (E) Observed (black) and modelled (red) dry (*T*_dry_) and wet (*T*_wet_) filter paper reference temperature kinetics used to calibrate the energy balance model. (F) Observed (black) and modelled (red) dry (*T*_dry_) and wet (*T*_wet_) filter paper reference temperature kinetics used to validate the energy balance model. Grey areas represent dark conditions.

In an enclosed environment (e.g. a phenotyping platform in a climate-controlled room), air mixing is important to prevent the creation of temperature and humidity gradients that could bias the interpretation of the results. Moreover, this air movement increases boundary layer conductance, which in turn improves the heat and water vapour exchange between the leaf and its surrounding environment, resulting in high contrast thermograms. If mixing is provided by a constant air flow, *g*_bh_ should be relatively constant and can be approximated as a unique value. If the air flow around the leaf is continuously changing (e.g. wind gust), *g*_bh_ can be calculated at a leaf scale from the aerodynamic theory as reviewed by Schuepp (1993):

$$\begin{array}{l} g_{bh}=0.00662{\left(u/l\right)}^{0.5} \end{array}$$ (8)

where *u* (m s^−1^) is the wind speed and *l* (m) is the characteristic dimension of the leaf. Using Equation 1 with a black and a white reference material, it is possible to validate *g*_bh_ values by comparing the predicted and observed temperature kinetics of the white reference material (Fig. 1D). Alternatively, Equation 1 can be solved for *g*_bh_ and used directly to predict variation in leaf boundary layer conductance using reference material with a similar shape and different optical properties:

$$\begin{array}{l} \begin{array}{l} g_{bh}=\frac{k_{2}\frac{dT_{2}}{dt}-k_{1}\frac{dT_{1}}{dt}-I_{s}\left(\alpha_{2}-\alpha_{1}\right)+2\sigma \left(\varepsilon_{2}{T_{2}}^{4}-\varepsilon_{1}{T_{1}}^{4}\right)}{2\rho C_{s}\left(T_{1}-T_{2}\right)}\; \end{array} \end{array}$$ (9)

This equation was used to calculate *g*_bh_ values from temperature kinetics of reference materials under the conditions of the experiments, and included in Equations 3 and 5.

#### Source of error in estimating stomatal conductance from leaf temperature

A common issue in interpreting thermograms using energy balance is the variation of parameter values (e.g. emissivity and leaf absorbance) between leaves that are difficult to control and to take into consideration. Large differences in leaf emissivity are not expected, with values ranging from 0.96 to 0.98 (maximum SD=0.01) for a range of different species reported in the literature (Chen, 2015). Differences in leaf absorbance and orientation between leaves are important as these influence the amount of light energy received by the leaf with consequence for their temperature. For example, an increase in leaf thickness can result in an increase in absorbance (α _1_) but can also affect the energy required to change the temperature of the leaf (*k*_1_). Even small variations (~ 0.01) in leaf emissivity (ε _1_) can impact temperature estimation due to the multiplication of large numbers when applying the Stefan–Boltzmann law (radiant energy emitted=εσ*T*^4^). Variation in leaf size can result in differences in boundary layer conductance, influencing the heat and water vapour exchange between the leaf and its surrounding environment. In this study, to simplify interpretation, leaves were placed horizontally under the lights and parameter values describing leaf properties were set based on a previous estimation in wheat (ε _1_, 0.96; α _1_, 0.85; *k*_1_, 700) and produced consistent predictions of leaf temperature kinetics using the two reference materials (black and white aluminium references). These parameters were considered constant over the leaf lamina and could have influenced the estimation of *g*_sw_. However, a sensitivity analysis of the energy balance model (Vialet-Chabrand and Lawson, 2019) showed that it is reasonable to expect that small variations in parameter values over the leaf lamina will have a limited impact on *g*_sw_.

## Materials and methods

### Plant material

A modern elite variety of spring wheat ‘Paragon’ (*Triticum aestivum* L.) was grown in a 20-well modular tray under well-watered conditions in peat-based compost (Levingtons F2S; Everris) for 1 week, and plants were vernalized for 10 weeks in a cold room at 5 °C. Plants were potted in 200 ml pots (*n*=10, one plant per pot) and moved to a controlled-environment chamber (Photon Systems Instruments, Brno, Czech Republic) with 300 µmol m^−2^ s^−1^ of light intensity (10 h/14 h) provided by white LEDs, temperature of 22/18 °C and a relative humidity (RH) of 50/65% (day/night). Plants were measured after 35 d of growth (Zadocks stage 25) on the upper most fully expended leaves. Hoagland’s nutrient solution (~100 ml per pot) was supplied weekly.

### Measuring equipment

Air RH and temperature (*T*_air_) were measured using a Rotronic HC2-SH sensor (Rotronic Instruments Ltd, Crawley, UK). Light intensity was measured with a Skye SP-210 PAR sensor (Skye Instruments Ltd, Llandrindod Wells, UK). A thermal camera (FLIR A655sc; FLIR system AB, Täby, Sweden) including an uncooled microbolometer detector (resolution, 640×480 pixels; spectral range, 7.5–14.0 µm; noise equivalent temperature difference, <30 mK) was used to capture thermograms. Illumination was provided by two identical LED light sources (LX601C, Heliospectra AB, Göteborg, Sweden) located on each side of the thermal camera. Photon flux received by the leaves was determined at 440 µmol m^−2^ s^−1^ with a quantum sensor (SKP 215; Skye Instruments Ltd) placed near the reference materials, and converted to energy (W m^−2^) using a radiation conversion factor measured with a spectroradiometer (model SR9910-PC, Macam Photometrics Ltd, Livingstone, UK).

### Reference materials

Two aluminium references with a thickness (l*) of 0.00095 m, a density (ρ*) of 2484 kg m^−3^, and a specific heat capacity (C* _p_) of 896 J kg^−1^ K^−1^ (resulting in *k*=l*ρ*C* _p_=2114 J m^−2^ K^−1^) were painted respectively in black and in white. Using an Ulbricht integrating sphere (built at the University of Essex), the absorbance of the aluminium references was measured at 0.05 (white reference) and 0.96 (black reference). Emissivity of the aluminium references was estimated at 0.96 for both. Emissivity was measured by placing a piece of black vinyl electrical tape with an emissivity of 0.96 on a part of the reference and by comparing the temperature reading of the thermal camera with that of the uncovered area. A thermocouple was placed between the electrical tape and the aluminium reference to validate the temperature reading from the thermal camera. Under dark conditions (to prevent differences in absorbed light intensity), emissivity of the area not covered with the tape was adjusted until the temperature matched with that from the area covered by the tape. This procedure was repeated under different ambient temperatures to detect any variations that could affect the temperature reading over a range of environmental conditions. It should be noted that reference material should be shaped to match the leaf characteristic dimension (*l*, Equation 8) and placed in the same orientation as the measured leaf.

### Temporal response model

The temporal responses of *g*_sw_ and *I*_g_ were assessed using the sigmoidal model published by Vialet-Chabrand and Lawson (2019). The initial lag time (φ) represented by the period with a quasi-absence of *g*_sw_ response after an environmental change and the time constant of the response (τ) were assessed using the following equation:

$$\begin{array}{l} \begin{array}{l} g_{sw}=\left(G+s_{l}\times t-g_{0}\right)\frac{e^{-e^{\frac{\;\varphi \;-t}{\tau }+1}}-e^{-e^{\frac{\;\varphi \;}{\tau }+1}}}{1-e^{-e^{\frac{\;\varphi \;}{\tau }+1}}}+g_{0} \end{array} \end{array}$$ (10)

Where *g*_0_ is the initial value of *g*_sw_ at *t*_0_=0, *G* is the steady-state target of *g*_sw_, and *s*_l_ the slope of the slow decrease/increase in *g*_sw_ (mol m^−2^ s^−2^). The same equation was used for *I*_g_ allowing comparison of φ and τ values between both interpretation methods of thermograms. Parameter values were estimated by minimizing the distance between observed and modelled data using the ‘optim’ function in R.

Appling the previous equation for each pixel of the thermogram values sometimes produced inconsistent results due to the diversity of stomatal responses and the measurement noise leading the curve-fitting algorithm to be trapped in local minima. Therefore, a simplified version of the model was applied simultaneously on the increasing and decreasing part of the *g*_sw_ response sharing the steady-tate target parameters (*G* and *g*_0_) adding more constrains to the curve fitting process:

$$\begin{array}{l} g_{sw}=G+\left(g_{0}-G\right)e^{-[max(0,t-\lambda )]/k} \end{array}$$ (11)

Any time *t* before λ resulted in *g*_sw_=*g*_0_, delaying the beginning of the exponential response controlled by the time constant *k*.

### Data analysis

The dynamic energy balance equations used here were implemented in R (www.r-project.org/) and are available as Supplementary Dataset S1 in a package called ‘leafNRG’. This package includes all the equations used and described here and enables the calculation of leaf boundary layer conductance, stomatal conductance, and transpiration, as well as the simulation of the dry and wet leaf temperature kinetics. It also includes a validation of the energy balance parameter (e.g. *g*_bh_) values by comparing predicted and observed temperature kinetics from reference materials.

## Results

### Prediction of ‘dry’ and ‘wet’ leaf temperature

The only environmental variables required to predict the ‘dry’ leaf temperature (*T*_dry_) were air temperature (*T*_air_, Fig. 1A) and light intensity (*I*_s_). The ‘wet’ leaf temperature (*T*_wet_) was predicted by including the water vapour gradient between the leaf and the atmosphere, calculated using the air RH (Fig. 1B). A smoothed spline function was used to transform discrete observations for *T*_air_ and RH in continuous variables (red lines) that could be used with the ODE solver. The ODE solver was initialized using the observed temperature at *t*=0. The difference between *T*_dry_ and *T*_wet_ reached >6 °C and followed distinct trajectories in response to changes in light intensity (Fig. 1C), as *T*_wet_ was influenced by the variations of *T*_air_ and RH. Rapid variations of *T*_air_ and RH were observed even in the dark due to oscillations in the environmental control unit (air conditioning unit and steam humidifier) of the room where measurements were conducted. The value calculated using Equation 9 for *g*_bh_ (0.826 mol m^−2^ s^−1^) and used to predict the ‘wet’ leaf temperature was validated by comparing the observed and predicted (Equation 3) white aluminium reference temperature kinetics (Fig. 1D, *R*^2^, 0.99; RMSE, 0.05 °C). The capability of the model to predict ‘wet’ and ‘dry’ reference temperature kinetics was validated using filter paper as a reference material. The ‘wet’ reference consisted of a piece of filter paper with the two ends submerged in water to ensure a constant supply of water to keep the material damp. A first data set was used to calibrate the model (Fig. 1E) using the gradient descent algorithm (‘optim’ function from R) tuning the parameter values (α _f_=0.15, *k*_f_=815s, *g*_bh_=0.006 m s^−1^) to minimize the distance between modelled and observed data (for both references). The set of parameter values was then applied to a different data set (Fig. 1F) with different environmental conditions to test the validity of the model. The predicted temperature kinetics showed high accuracy to reproduce the observed data.

### Interpretation of thermograms

**Fig. 2.**
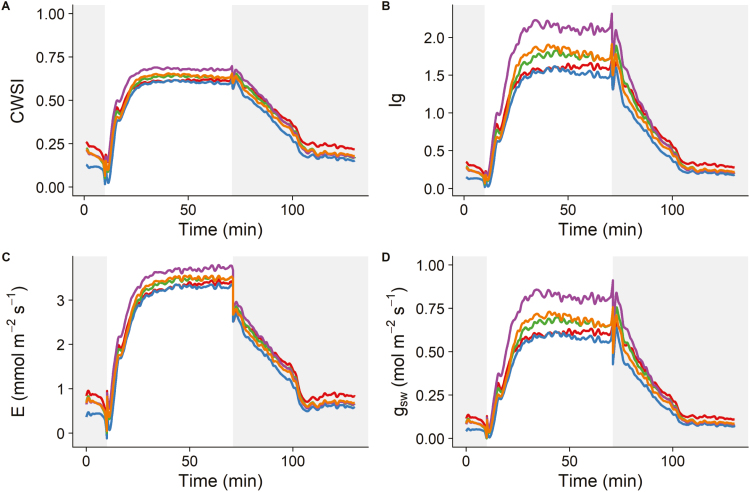
Estimated (A) crop water stress index (CWSI), (B) conductance index (*I*_g_), (C) transpiration rate (*E*), and (D) stomatal conductance to water vapour (*g*_sw_) in wheat (Paragon) after a step increase in light intensity from dark to light (0 to 440 µmol m^−2^ s^−1^, 1 h) and a step decrease from light to dark (1 h). Grey areas represent dark conditions.

Temperature kinetics of five wheat leaves to a step increase (0 µmol m^−2^ s^−1^ to 440 µmol m^−2^ s^−1^ maintained for 60 min) and step decrease (440 µmol m^−2^ s^−1^ to 0 µmol m^−2^ s^−1^ maintained for 60 min) in light were extracted from thermograms and are presented in Fig. 1C. After the light intensity was increased, leaf thermal kinetics followed those of *T*_dry_, as stomata were closed, limiting evaporative cooling. As stomata opened, leaf temperature kinetics moved toward *T*_wet_ (without reaching it), and stabilized to an intermediate value between *T*_dry_ and *T*_wet_. Conversely, when light was returned to zero, temperature slowly moved back toward *T*_dry_. Using *T*_dry_ and *T*_wet_, CWSI and *I*_g_ were calculated using Equations 1 and 2, respectively, and displayed a similar pattern of variation (Fig. 2A, B), although on a different scale. Both stress indices exhibit a rapid exponential increase after the light level was increased and slowly returned to the initial temperatures under dark conditions. Using Equation 4, *E* was calculated and displayed a an exponential increase similar to the stress indices under illumination (Fig. 2C). However, when the light was switched off, a sudden drop in *E* of ~1 mmol m^−2^ s^−1^ was observed, that was mainly due to the rapid decrease in leaf temperature and VPD. This sudden decrease in *E* was not reflected by the stress indices that related to *g*_sw_. Using Equations 6 and 7, *g*_sw_ was calculated and displayed a similar pattern of variation to the stress indices (Fig. 2D); however, there were larger differences between leaves than those observed with the CWSI. Despite the differences in scale when using different methods to interpret thermograms, the parameter values describing the temporal responses of *g*_sw_ and *I*_g_ (Equation 10) were not significantly different (*P*>0.05). On average (*n*=5 leaves), the initial lag time (φ) was 2.1±0.1 min when estimated from *g*_sw_ and 2.3±0.2 min when estimated from *I*_g_. The time constant of the response (τ) was 5.3±0.3 min when estimated from *g*_sw_ and 5.2±0.2 min when estimated from *I*_g_.

### Impact of stomatal limitation on leaf temperature

**Fig. 3.**
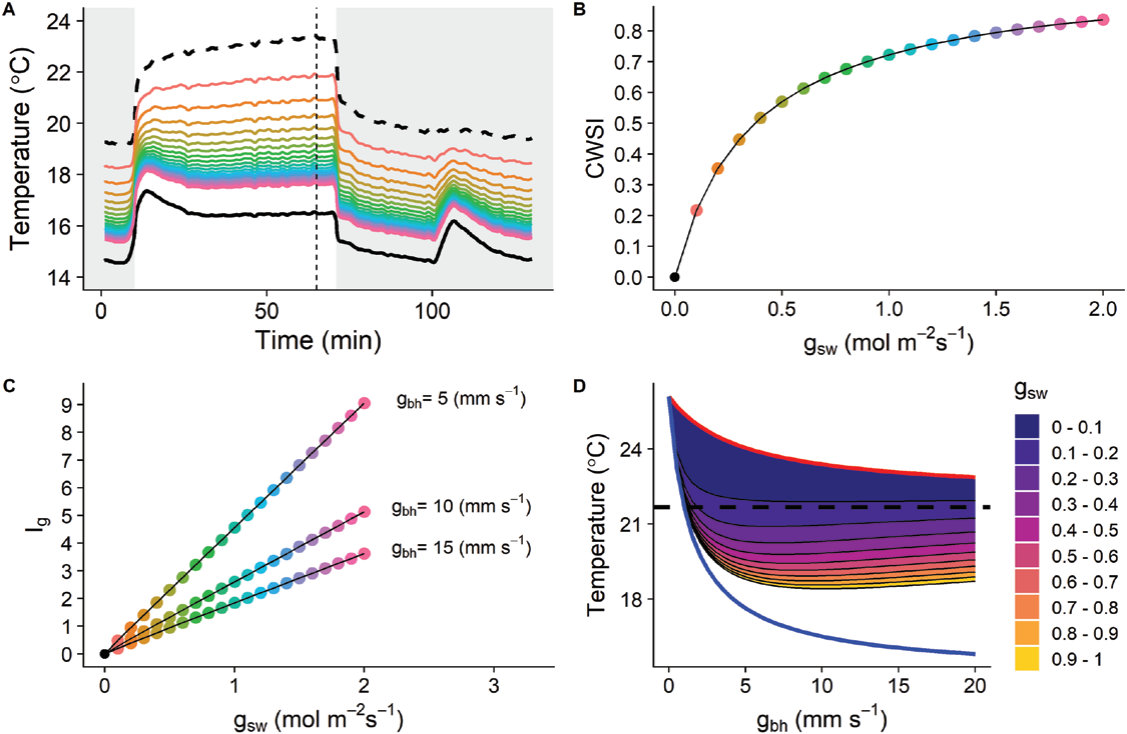
(A) Modelled temporal kinetics of leaf temperature for stomatal conductance to water vapour (*g*_sw_) ranging from 0.1 mol m^−2^ s^−1^ to 2 mol m^−2^ s^−1^ (step: 0.1 mol m^−2^ s^−1^, coloured lines) and predictions for a non-transpiring (black dashed line) and an unlimited stomatal conductance leaf (black continuous line). (B) Crop water stress index as a function of *g*_sw_. (C) Conductance index (*I*_g_) as a function of *g*_sw_ for different one-side boundary layer conductance (*g*_bw_). The coloured lines (A) and dots (B, C) correspond, respectively, to the same *g*_sw_ values. (D) Relationship between leaf temperatures simulated for different *g*_sw_ and *g*_bw_ illustrating the changes in scale between the dry (red line) and wet (blue line) references when *g*_bw_ varies. The black dashed line represents the air temperature. The vertical dashed line in (A) indicates the time (65 min) at which the relationships in (B), (C), and (D) were estimated.

Different leaf temperature kinetics were predicted using constant *g*_sw_ values ranging from 0 to 2 mol m^−2^ s^−1^ (Fig. 3A) to describe the relationship between CWSI, *I*_g_, and *g*_sw_ (Fig. 3B, C). At *g*_sw_ values <0.5 mol m^−2^ s^−1^, a small variation in *g*_sw_ almost linearly correlates with a decrease in leaf temperature as part of the limitation on evaporative cooling was removed. However, as *g*_sw_ increased above 0.5 mol m^−2^ s^−1^, the effect on leaf temperature diminished and larger variations in *g*_sw_ values were required to alter the temperature. This behaviour resulted in the non-linear relationship displayed between CWSI and *g*_sw_ (Fig. 3B). Under controlled conditions, *I*_g_ and *g*_sw_ were linearly related (Fig. 3C); however, it is important to note that different *g*_bw_ values (e.g. different air mixing around the leaf) would influence conduction and leaf evaporative cooling and result in a change in scale for *I*_g_. Figure 3D illustrates the change in scale between the ‘wet’ and ‘dry’ reference temperatures in response to *g*_bh_ variation. As *g*_bh_ increased, the ‘wet’ and ‘dry’ temperature showed an exponential decrease with different magnitude and slope. The different *T*_leaf_ simulated for a range of *g*_sw_ and *g*_bh_ values do not follow the same kinetic response as the references, explaining the resulting variations in *I*_g_. In general, *I*_g_ and *g*_sw_ were linearly correlated for a given *g*_bh_, but the slope of the relationship can change depending of the environmental conditions (*I*_s_, *T*_air_, RH, *g*_bh_, etc.), therefore limiting the interpretation of *g*_sw_ differences.

### Spatial heterogeneity of stomatal behaviour

**Fig. 4.**
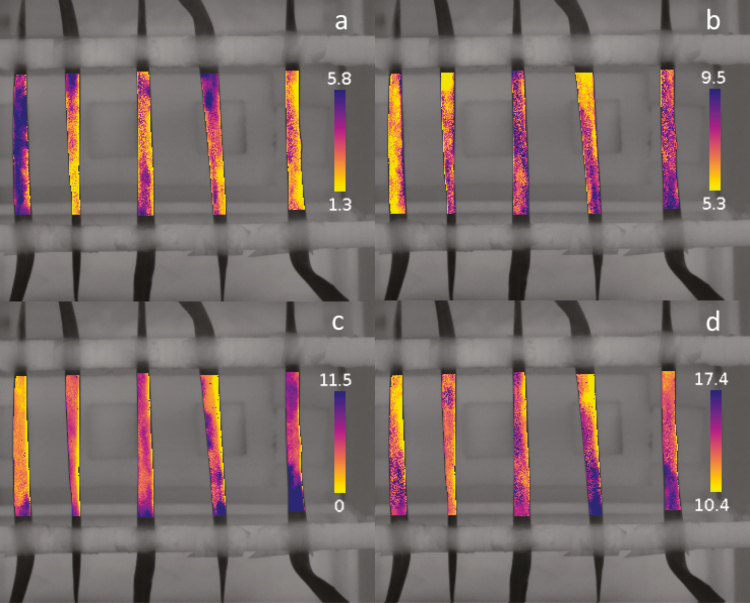
Spatial representation of the speed of stomatal response for a step increase (a, b) and decrease (c, d) in light intensity. The speed of stomatal response is described by a lag time (λ; a, c) and a time constant (*k*; b, d) derived from Equation 11. The colour scales represent an interval that contains 95% of all the values (min). Each pixel represents an area of ~0.6 mm^2^ (1 pixel per 0.77 mm).

**Fig. 5.**
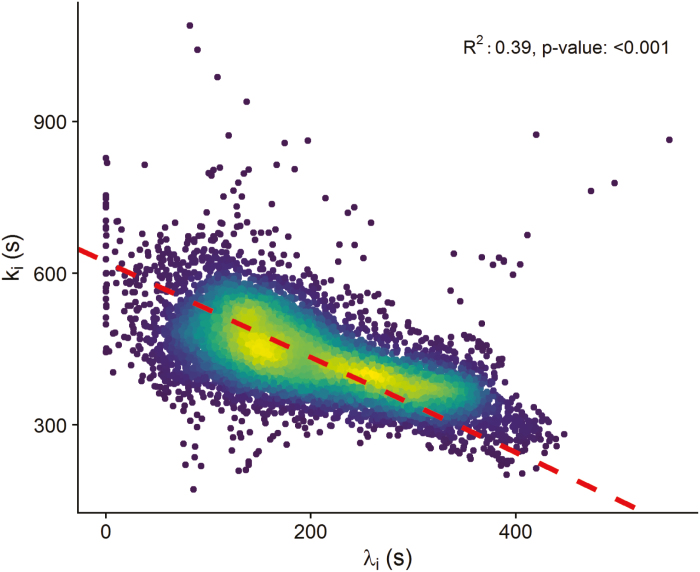
Relationship between the time lag (λ _i_, s) and time constant (*k*_i_, s) estimated using Equation 11 and describing the speed of stomatal responses for a step increase in light intensity of five wheat leaves. The red dashed line represents a standard major axis regression line. The colour gradient represents the density of points, with yellow being the highest density.

Solving Equations 6 and 7 on each pixel (1 pixel represented ~0.6 mm^2^ and ~40 stomata) of the collected thermograms produced a series of pictures representing the spatial and temporal heterogeneity of the stomatal responses. Supplementary Video S1 emphasizes how patterns of *g*_s_ behave differently over the leaf lamina in response to a step increase and decrease in light intensity. The temporal response of *g*_sw_ was described by two parameters (Equation 11) that were represented for each pixel of the thermogram (Fig. 4). In general, the results revealed a large spatial heterogeneity for both parameters and lighting conditions. Interestingly, values of the initial time lag and time constant observed after a step increase in light intensity were significantly correlated (Fig. 5, *P*<0.001, *R*^2^ 0.39) in all leaves with a different degree of correlation for each leaf. This correlation highlighted an interesting behaviour where areas of the leaf lamina with a large initial lag time were also the fastest to respond, resulting globally in a similar speed of response. There was no correlation between the rapidity of the stomatal response and the level of *g*_sw_ reached during the experiment.

## Discussion

Simplifying biological interpretation of thermograms captured under a dynamic environment is key to increased use of thermal imaging to study plant water use. Stress indices estimated from the simulated ‘dry’ and ‘wet’ temperatures suffered from different limitations that reduce their application in the field. CWSI was not linearly related to either *E* or *g*_sw_, and did not allow plants with different *g*_sw_ to be clearly identified, potentially impairing plant selection. Differences in *g*_sw_ especially when *g*_sw_ reaches high values and when *g*_bw_ becomes the main limiting factor are sensitive to small temperature differences, which are not apparent when using CWSI. The dependence of *I*_g_ values on the current environmental conditions limits this method to steady-state conditions. These limitations compared with methods using leaf energy balance remind us that stress indices are only a proxy for the biological responses and should be interpreted with care. It is interesting to note that under controlled conditions, the rapidity of stomatal responses was comparable when estimated from *I*_g_ and *g*_sw_ (despite the difference in magnitude), which suggests that *I*_g_ could be used as a proxy to estimate the rapidity of *g*_sw_ responses. Based on our results, future research using *I*_g_ should consider the use of leaf replicas with known conductance, as described in Vialet-Chabrand and Lawson (2019), as a way to continuously relate *I*_g_ and *g*_sw_. The conductance of such leaf replicas can be controlled by the density of pores over the surface which determines diffusion of water to the atmosphere. As *I*_g_ and *g*_sw_ are linearly related in most cases (Guilioni *et al.*, 2008), using a minimum of three leaf replicas with different *g*_sw_ (covering the range of values expected from the targeted species) along with ‘wet’ and ‘dry’ references will enable *I*_g_ values to be calculated for the leaf replicas that will directly relate to a known *g*_sw_. Using this method solves the problem of scale changes for *I*_g_ and should enable continuous estimatation of *g*_sw_ without complex equipment or calculations.

Compared with previous works using reference materials (Leinonen *et al.*, 2006; Guilioni *et al.*, 2008; Maes *et al.*, 2016; Jones *et al.*, 2018), adding a second reference material with different optical properties enables validation of parameter values from the energy balance equations by comparing observed and modelled temperature kinetics and the calculation of *g*_bw_ using Equation 9. *g*_bw_ is a major determinant of the energy budget, and small errors in its estimation can result in large errors when determining *g*_sw_, especially when *g*_bw_ values are low. Empirical relationships between *g*_bw_ and wind speed that are often used in field experiment (Jones, 2013) can be calibrated and validated in the future using reference material temperature kinetics and Equation 9. Placing reference materials at different locations in the field could help to take into account spatial heterogeneity in micro-climatic conditions when imaging large areas and account for local variation in *g*_bw_. Moreover, several references could be placed at different angles to mimic the shape of a plant (without an excessive cost) to account for the distribution of leaf angles in the canopy in the calculation of *g*_sw_ and *g*_bw_. Using a Monte Carlo approach, it should be possible to produce a distribution of *g*_sw_ and *g*_bw_ values by including the distribution of parameter values representing the diversity of the canopy rather than single leaf values. The potential of the method described herein to interpret thermal kinetics in a dynamic environment is vast and could lead to several applications in the future such as predicting potential canopy transpiration, a key parameter for irrigation management.

In this study, we have introduced a simplified method to solve energy balance equations for *E* and *g*_sw_ under dynamic environmental conditions. In contrast to Vialet-Chabrand and Lawson (2019), this method allows *E* and *g*_sw_ to be determined without having to use an ODE solver and a specific model to describe *g*_sw_ variations. When applied to each pixel of a thermogram, the spatial heterogeneity displayed by the parameter values describing the temporal response of *g*_sw_ stresses the importance of the temporal dimension to explain the spatial patterns of *g*_sw_ observed in this study. Previously, Haefner *et al.* (1997) used a computer model of stomatal functioning to simulate ‘patchy’ stomatal behaviour. They observed that simulations performed with a similar temporal behaviour for all stomata required a considerable amount of time for the conductance of individual patches to diverge enough to produce a patchy pattern (Mott and Buckley, 2000). This suggested the need for heterogeneous temporal responses of *g*_sw_ to produce transient patchy patterns like those observed after a change in environmental conditions. After a step increase in light intensity, stomata displayed spatial patterns of initial lag time, with patches of stomata starting to open whilst other stayed closed for a period of up to ~6 min. This partial stomatal opening causes an immediate increase in the whole-leaf rate of water loss without directly affecting the rate of water supply to the leaf. The variability in the delay of response following a change in environmental conditions could be a mechanism to balance the increase in evaporative demand throughout the leaf and maintain the xylem water potential until the hydraulic conductance adjusts to the new conditions. The correlation between the delay and the rapidity of the response suggested than stomata with greater lag times were faster to respond, which may be due to the higher water availability at this phase of the response compared with the initial conditions. Our hypothesis is that water is a limited resource that is shared across the entire leaf lamina and is compatible with previous observations by Buckley and Mott (2000) who used two independent gas exchange systems to monitor two patches in a wheat leaf. When light was turned off in one of these patches, an increase in *g*_sw_ was observed in the other illuminated patch. This is consistent with the idea that the change in water loss and therefore xylem water potential in the non-illuminated patch could result in an increase in water available in the illuminated patch, allowing higher stomatal aperture. In future studies, our approach could be coupled with an analysis of the leaf anatomy to elucidate the cause of the patchy stomatal behaviour.

The methods presented herein pave the way for new developments in thermal imaging and applications in plant physiology at different scales of integration. Here, we have described how thermal indices that are generally used in the field to estimate stomatal responses could lead to incorrect interpretations, for example in monitoring plant water status. Furthermore, we highlight how variations in the rapidity of stomatal response over the leaf lamina can drive patchy stomatal behaviour following a step change in light intensity and influence the fine regulation of water use to maintain leaf water potential. The fact that stomata in some areas of the leaf are not responding as rapidly or with the same magnitude, following changes in light intensity (similar to those observed during sun-flecks), could provide a mechanism to limit water loss, but also lead to inefficiencies in assimilation rate, that when scaled up could be economically important.

## Supplementary data

Supplementary data are available at *JXB* online.

Dataset S1: ‘leafNRG_0.1.0.tar.gz’ is an R package implementing the leaf energy balance equations described in the manuscript with an example data set.

Video S1: the video entitled ‘Patchy_Behaviour.mp4’ shows the temporal and spatial heterogeneity of stomatal conductance over the leaf lamina of five wheat plants in response to a step increase and decrease in light intensity.

erz573_suppl_supplementary_dataset_S1

erz573_suppl_supplementary_video_S1
